# The influence of cooling on biomechanical time since death estimations using ovine brain tissue

**DOI:** 10.1007/s00414-024-03267-3

**Published:** 2024-06-20

**Authors:** Johann Zwirner, Pavithran Devananthan, Paul D. Docherty, Benjamin Ondruschka, Natalia Kabaliuk

**Affiliations:** 1 Institute of Legal Medicine, University Medical Center Hamburg-Eppendorf, Hamburg, Germany; 2 Department of Oral Sciences, University of Otago, Dunedin, New Zealand; 3 Department of Mechanical Engineering, University of Canterbury, Christchurch, New Zealand; 4 Biomolecular Interaction Centre, University of Canterbury, Christchurch, New Zealand

**Keywords:** Biomechanics, Brain, Rheometry, Post-mortem interval, Time since death estimation

## Abstract

The significance of biomechanical analyses for forensic time since death estimations has recently been demonstrated. Previous biomechanical analyses successfully discriminated post-mortem brain tissue from tissue with a post-mortem interval of at least one day when held at 20 °C. However, the practical utility of such analyses beyond day one at 20 °C was limited. This study investigates the storage, loss, and complex shear modulus of various brain regions in sheep stored at 4 °C in 24-hour intervals over four days post-mortem using rheometry tests. The aim is to identify the critical biomechanical tissue property values to predict post-mortem time and assess the temperature sensitivity of the rheometry method by comparing results to recent findings at 20 °C. Thirty sheep brains were examined, including the frontal lobe, parietal lobe, anterior and posterior deep brain, superior colliculi, pons, medulla, and cerebellum. Rheometry tests were conducted, and receiver operator characteristic analyses were employed to establish cut-off values. At 4 °C storage, all investigated biomechanical properties of the examined brain regions remained stable for at least one day post-mortem. Using cerebellar samples stored at 4 °C, a post-mortem interval of at least two days could be determined with excellent diagnostic ability. Complex shear modulus values below 1435 Pa or storage modulus values below 1313 Pa allowed prediction of two or more days post-mortem. Comparisons between 4 °C and 20 °C revealed brain region-specific results. For instance, the complex shear moduli of the anterior deep brain at 4 °C were significantly higher on all individual testing days when compared to 20 °C. In contrast, the combined medulla and pons samples were similar on each day. Rheometry testing of brain tissue consistently stored at 4 °C since death proved valuable for forensic time since death estimations starting from two days after death.

## Introduction

The value of post-mortem biomechanical analyses of brain tissue for forensic time since death estimation has recently been demonstrated using ovine tissue [1, 2]. By employing cerebellar samples, it was possible to distinguish immediately post-mortem samples from those stored at 20 °C for one to four days with a sensitivity and specificity of 90% and 92%, respectively [2]. However, the inability to distinguish between biomechanical properties of brain tissue across days two to four limited the forensic value of the proposed rheological analyses of brain tissue at 20 °C. Lower temperatures generally prolong the degradation of biological tissues [3]. Hence, at lower temperatures, biomechanical analyses of brain tissue for time since death estimations might be promising even beyond day one.

From both a forensic and broader scientific perspective, it is of interest to compare the previous observations at 20 °C to a storage temperature of 4 °C, using the same setup. From a practical perspective, 20 °C is a sensible assumption when the body is located in an interior temperature controlled environment. In the morgue, cadavers are commonly stored at 4 °C between admission and autopsy to limit degradation effects. For forensic time since death analyses, an ambient temperature of 4 °C is relevant when bodies are found outside during the colder months of the year. Moreover, the temperature of 4 °C corresponds to the year-round water temperature at the bottom of stagnant waters, such as lakes where bodies (or heads in dismemberment cases) are occasionally disposed after homicides [4]. For biomechanical basic research, brain samples are commonly stored at approximately 4 °C between retrieval and further use to prevent degradation [5–9].

Post-mortem analyses of the biomechanical properties of brain tissue over a time frame of several days after death remain scarce [2, 10]. This scarcity is due to the fact that tissue degradation is commonly undesired for biomechanical analyses. Predominantly, biomechanical research on brain tissue aims at obtaining lifelike properties to allow for an accurate simulation of load deformation properties in head impact scenarios [11]. Hence, researchers strive to keep the post-mortem intervals (PMIs) to a minimum, with a recommendation to stay within a few hours between death and biomechanical analysis [12].

Measuring the mechanical properties of soft tissues is challenging due to their malleability, requiring specialized devices [2, 13, 14]. Rheology is commonly employed to assess these properties, focusing on viscoelastic characteristics in tissues like the brain [10, 15–17], liver [18–21], kidney [21, 22], and muscle [23]. Oscillatory rheology efficiently measures a sample’s storage modulus (S_mod_), loss modulus (L_mod_), and complex shear modulus (CS_mod_). The S_mod_ indicates elasticity, reflecting the material’s energy storage ability, while the L_mod_ indicates viscosity, signifying energy dissipation characteristics. The CS_mod_ combines both, representing the material’s resistance to deformation [24]. With tissue decomposition, there’s a progressive loss in deformation resistance, leading to a simultaneous reduction in all three moduli [2].

This study aimed to provide forensically and scientifically relevant biomechanical data on brain tissue over a time frame of four days post-mortem at 4 °C. Emphasis was placed on establishing cut-off values that link the biomechanical properties to indicative points in time post-mortem based on their practical relevance for forensic investigators. The hypothesis posits that, compared to the post-mortem storage temperature of 20 °C, storage at 4 °C improves the resolution beyond day one of time since death classification possible from rheological analysis.

## Materials/methods

### Tissue collection and preparation

Ovine full brains (*n* = 30), including the medulla, were retrieved within two hours of sacrifice. The sheep were sacrificed for meat by throat slitting, and the brains were considered a waste product. Therefore, no ethical approval was required for this study.

**Fig. 1:**
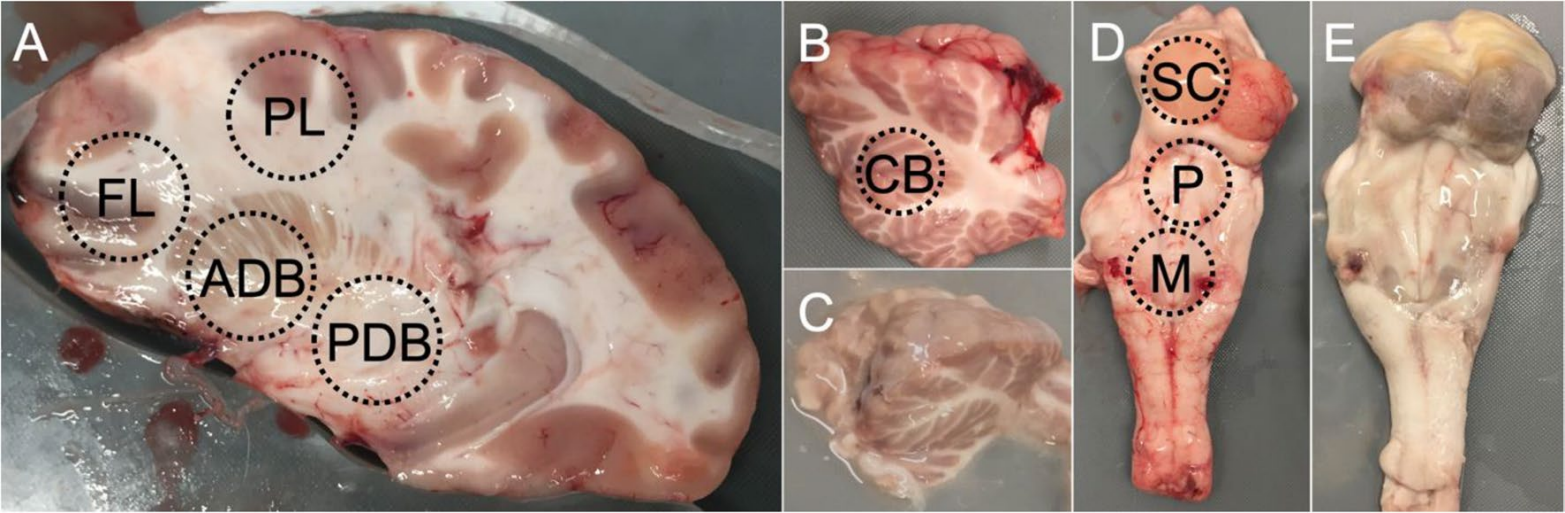
**(A)** Cerebral slice including punching regions with a post-mortem interval (PMI) of two days at 4 °C. **(B)** Cerebellar piece including punching region with a PMI of two days at 4 °C. **(C)** Comparative cerebellar piece with a PMI of two days at 20 °C. **(D)** Midbrain-pons-medulla piece including punching regions with a PMI of two days at 4 °C. **(E)** Comparative midbrain-pons-medulla piece with a PMI of two days at 20 °C. ADB, anterior deep brain; CB, cerebellum; FL, frontal lobe; M, medulla; P, pons; PDB, posterior deep brain; PL, parietal lobe; SC, superior colliculi

Immediately after retrieval, the brains were submerged in phosphate-buffered saline (PBS) to rinse off debris from blood and the retrieval process. Subsequently, the brains were placed in containers with freshly prepared PBS at 4 °C until mechanical testing. The day 0 tests were completed within four hours after sacrificing. The maximum sample size per brain region and testing day was 12 and 6 for paired samples (e.g., frontal lobe samples) and unpaired samples (e.g., pons samples), respectively. For each paired brain region, an equal ratio of left and right samples was ensured. Just before mechanical testing, samples were punched from eight different brain regions using a biopsy punch with a diameter of 10 mm. For cerebral samples, sagittal brain slices were cut with a microtome blade and an 8-mm-thick laser-cut acrylic stencil. This process created a medial brain slice, including the deep brain structures, from which frontal lobe (FL), parietal lobe (PL), as well as anterior (ADB) and posterior deep brain (PDB) samples were punched according to Fig. 1A. Similarly, the stencil was used to produce lateral slices of the cerebellum, from which a sample (CB) was punched (Fig. 1B). From the midbrain area, the left superior colliculus (SC) sample was punched from posterior to anterior (Fig. 1D). Likewise, pons (P) and medulla (M) samples were prepared (Fig. 1D). After punching, the sample height was adjusted to 5 mm using a customized mold and a microtome blade. Each day of testing utilised new samples, no sample was tested twice. The comparative data for samples stored at 20 °C (Fig. 1C, E) were taken from our previous work [2], for which the exact same testing protocol was used.

### Biomechanical testing

**Fig. 2:**
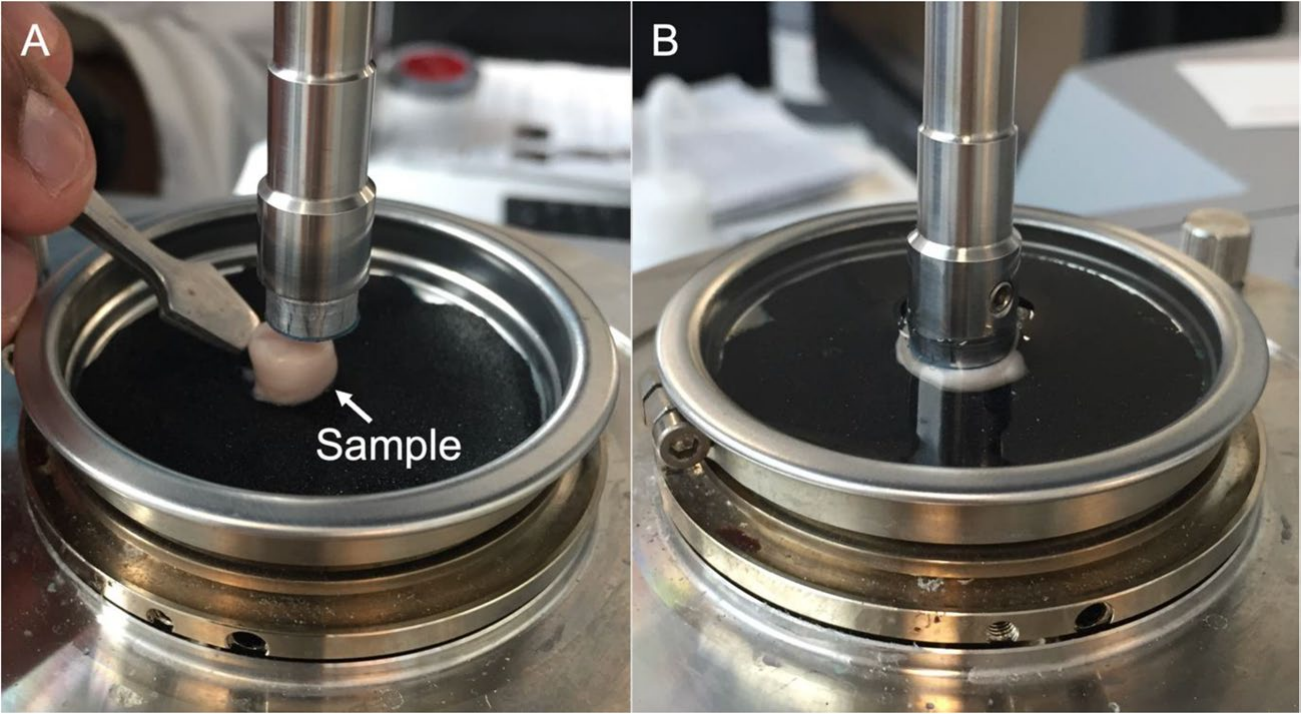
**(A)** Positioning of the cylindrical samples between the sandblasted contact surface of the rotating shaft and the stable base plate covered with sandpaper. **(B)** Tests were performed in phosphate-buffered saline at 20 °C

Biomechanical testing was conducted using a rheometer (MCR302; Anton Paar, Graz, Austria). The tissues were placed into the apparatus under an axial preload of 0.1 N to ensure contact surface adhesion and were given 100 s to relieve any residual stresses. To reduce slippage, sandpaper was inserted between the base plate of the rheometer and the samples and the contact surface of the measuring tool was sandblasted (Fig. 2A).

The rheometer was calibrated using PBS and all tests were performed in PBS at 20 °C (Fig. 2B). A peak angular shear strain of 0.03 [rad] at 3 Hz, with a continued 0.1 N compression force, was applied for a total of 50 cycles. This approach followed the methodology outlined in our previous study [2].

### Data analysis

The measured S_mod_, L_mod_, and CS_mod_ of the tissues were analysed using Microsoft Excel Version 16.74 (Microsoft Corporation, Redmond, USA) and GraphPad Prism version 9 (GraphPad Software, La Jolla, USA) for statistical analyses and data visualization. The Kolmogorov-Smirnov test was employed to assess the data for Gaussian distribution. Depending on the normality distribution, either ordinary one-way ANOVA tests, including Tukey’s multiple comparisons, or Kruskal-Wallis tests, including Dunn’s multiple comparisons, were applied when at least three groups were present. For cases with only two groups, parametric t-tests and nonparametric Mann-Whitney U-tests were applied for parametric and nonparametric data, respectively. P-values ≤ 0.05 were considered statistically significant.

Side comparisons between the cerebral hemispheres of different brains, as well as a comparison between the M and P samples, were conducted to determine whether the respective samples could be pooled for further analyses. The data from all corresponding samples of all tested days were then compared. Receiver Operator Characteristic (ROC) curves with a 95% confidence interval were generated from the group comparisons between the testing days, focusing on significant differences from day 0. Specifically, all testing days with no significant difference from day 0, including day 0, were grouped against all remaining testing days starting from the first day to significantly differ from day 0. The cut-off values with the highest positive likelihood ratio were selected from the computed results. In cases where the highest positive likelihood ratio was present more than once, the one with the highest sensitivity value was chosen.

## Results

### Day 0 comparisons

**Fig. 3:**
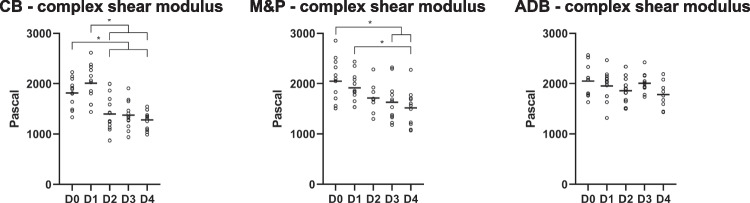
Representative plot graphs (minima to maxima with the median as a horizontal black line) of the complex shear modulus of three different sampling regions over five testing days are shown. The cerebellum (CB) shows a steep decrease between day one and day two while the decrease of the combined medulla and pons samples (M&P) is gradual. The anterior deep brain (ADB) samples showed constant values throughout the testing days. D, day; *, statistically significant difference (p-value < 0.05)

Pooling of left and right hemisphere samples from different brains, as well as the M&P samples, was performed as no statistically significant differences were observed across the hemispheres. A test matrix of all successfully tested samples per day and region and a graphical representation of all statistically significant differences among the tested brain regions and biomechanical properties is provided in Tables 1 and 2. The trends of the investigated biomechanical properties over the five testing days were region-specific, exhibiting differences in rheological behaviour (Fig. 3). In particular, the CB samples showed an abrupt decrease to a plateau; the M&P samples had a gradual decrease, and ADB samples showed minimal degradation of shear modulus.

None of the brain regions showed statistically significant differences between day 0 and day 1 samples for any of the investigated biomechanical properties. The ADB and PDB samples revealed no significant differences for any of the comparisons between day 0 and 4. Day 2 samples were significantly different from day 0 samples for the S_mod_ of the PL (*p* = 0.035) and the CB (*p* = 0.008), as well as the CS_mod_ of the CB (*p* = 0.009). Day 3 samples exhibited significant differences from day 0 for the S_mod_ and CS_mod_ of the CB (S_mod_: *p* = 0.005; CS_mod_: *p* = 0.005) and M&P samples (S_mod_: *p* = 0.046; CS_mod_: *p* = 0.046). For the L_mod_, the day 0 and 3 comparison only revealed statistically significant differences for the CB (*p* = 0.049). On day 4, samples significantly differed in S_mod_ and CS_mod_ from day 0 samples for the PL (S_mod_: *p* = 0.012; CS_mod_: *p* = 0.027), CB (S_mod_: *p* = 0.004; CS_mod_: *p* < 0.001), M&P (S_mod_: *p* = 0.004; CS_mod_: *p* = 0.007), and the SC (S_mod_: *p* = 0.033; CS_mod_: *p* = 0.049). Day 4 and day 0 samples of the FL also significantly differed in their S_mod_ (*p* = 0.024). L_mod_ was only significantly different for the CB samples between days 0 and 4 (*p* = 0.024).

**Table 1:** A test matrix including the number of successfully tested samples per day and region is depicted. ADB, anterior deep brain; CB, cerebellum; D, day; FL, frontal lobe; M&P, medulla and pons; PDB, posterior deep brain; PL, parietal lobe; SC, superior colliculi

Region	D0	D1	D2	D3	D4
**FL**	12	12	12	11	12
**ADB**	12	12	12	12	12
**PDB**	12	11	11	12	12
**PL**	12	12	12	12	12
**CB**	12	12	12	12	12
**M&P**	12	12	10	12	12
**SC**	6	6	5	6	6

**Table 2:**
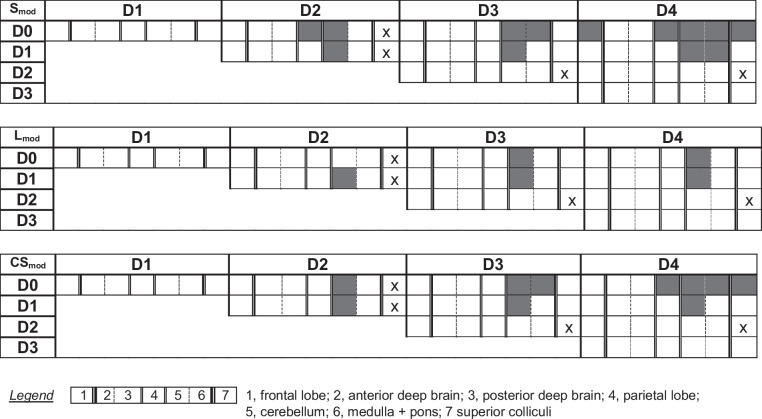
Graphical depiction of statistically significant differences of the storage (S_mod_), loss (L_mod_) and complex shear modulus (CS_mod_) values are depicted. Statistical comparisons with p-values below 0.05 are marked with grey color. D, day; x, no comparison due to insufficient number of samples (n < 6)

### Comparisons between the remaining testing days

Day 1 values of the CB samples were significantly different in all tested biomechanical properties from day 2 (S_mod_: *p* < 0.001; CS_mod_: *p* < 0.001; L_mod_: *p* = 0.005), day 3 (S_mod_: *p* < 0.001; CS_mod_: *p* < 0.001; L_mod_: *p* = 0.001), and day 4 (S_mod_: *p* < 0.001; CS_mod_: *p* < 0.001; L_mod_: *p* < 0.001). Additionally, the day 1 and 4 comparison was significantly different for the S_mod_ of the M&P samples (*p* = 0.046).

### Receiver operator characteristic curves

**Fig. 4:**
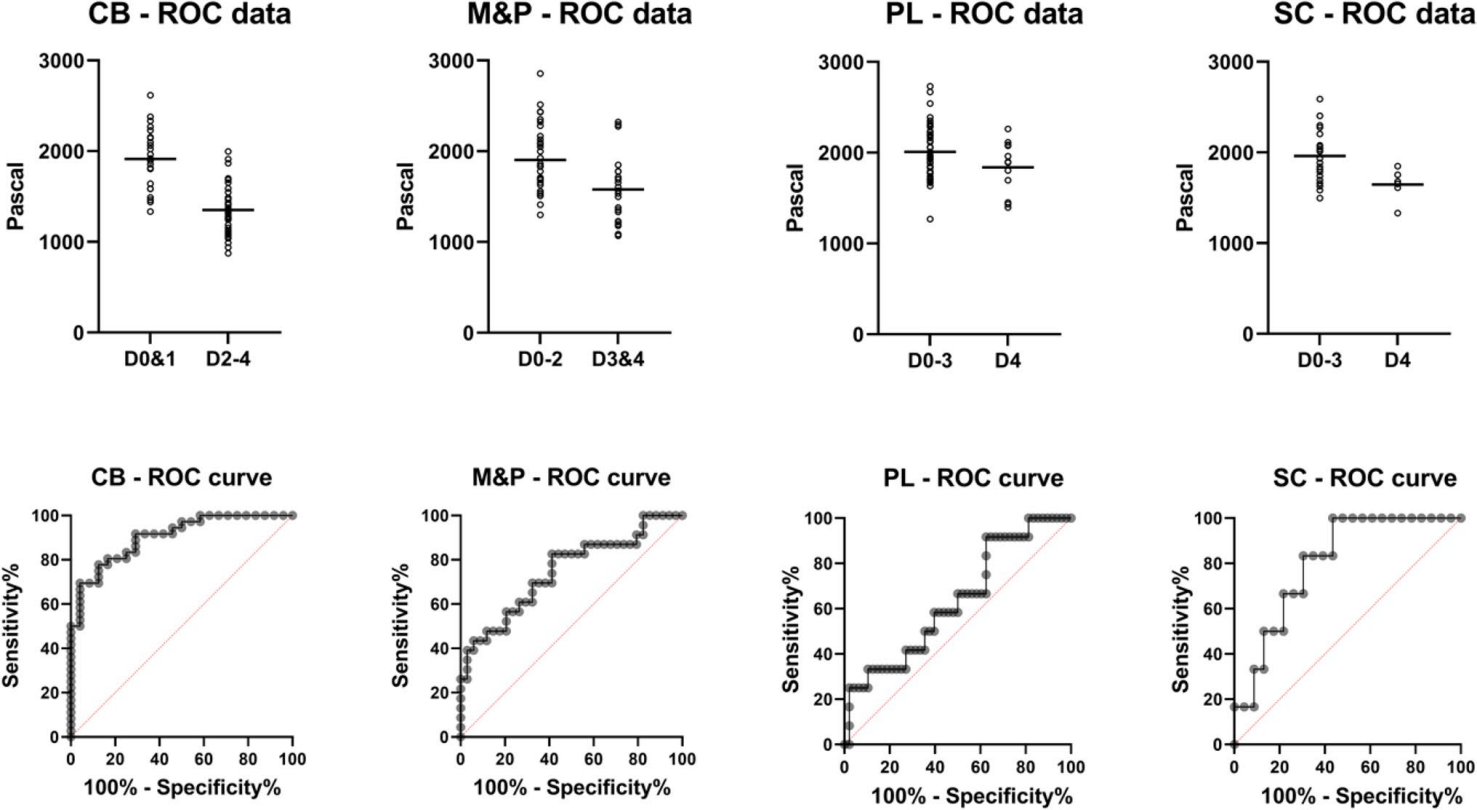
The aligned dot plot graphs (minima to maxima with the median as a horizontal black line) and corresponding receiver operator characteristic (ROC) curves of the complex shear moduli of the four different brain regions that revealed significant differences throughout the five-day testing interval are shown. CB, cerebellum; M&P, medulla and pons; PL, parietal lobe; SC, superior colliculi. D, day

The results of the ROC curve analyses are detailed in Table 3. Representatively, the grouping and curves for the CS_mod_ data are illustrated in Fig. 4. A PMI of at least two days could be determined with fair to excellent diagnostic ability using PL and CB samples. With M&P and CB samples, a PMI of at least three days could be diagnosed with fair to good diagnostic ability. A PMI of at least four days could be detected with poor to good diagnostic ability using FL, SC and PL samples.

**Table 3:** The cut-off values of the investigated biomechanical properties with the best positive likelihood ratios of the ROC curve analyses are depicted. AUC, area under the curve; CB, cerebellum; CI, confidence interval; FL, frontal lobe; M&P, medulla and pons; PL, parietal lobe; PMI, post-mortem interval; SC, superior colliculi

Property	Region	AUC	PMI [days]	Cut-off value [MPa]	Sensitivity [%]	95% CI [%]	Specificity [%]	95% CI [%]	Likelihood ratio
**S**_**mod**_	PL	0.757	≥ 3	< 1593	19	9.75–35.03	96	79.76–99.79	4.67
	CB	0.901	≥ 2	< 1313	67	50.33–79.79	96	79.76–99.79	16.00
	M&P	0.753	≥ 3	< 1301	39	22.16–59.21	97	85.08–99.85	13.30
	FL	0.720	≥ 4	< 1263	33	13.81–60.94	98	88.89–99.89	15.67
	SC	0.819	≥ 4	< 1491	33	5.92–70.00	96	71.01–99.78	7.67
**L**_**mod**_	CB	0.814	≥ 3	< 415	17	6.68–35.85	97	85.83–99.86	6.00
**CS**_**mod**_	CB	0.903	≥ 2	< 1435	69	53.14–82.00	96	79.76–99.79	16.67
	M&P	0.747	≥ 3	< 1395	39	22.16–59.21	97	85.08–99.85	13.30
	PL	0.635	≥ 4	< 1543	25	8.89–53.23	98	89.10–99.89	12.00
	SC	0.804	≥ 4	< 1656	50	18.76–81.24	87	67.87–95.46	3.83

### Comparison of 4 and 20 °C data

The comparison of data with storage temperatures of 4 and 20 °C revealed region-specific results (Table 4). For example, the CS_mod_ of the ADB was significantly different for each individual testing day, while for the M&P samples, it remained indistinguishable throughout all testing days.

**Table 4:** Significant p-values for the statistical comparisons of the complex shear moduli per testing day between storage temperatures of 4 and 20 °C are shown. ADB, anterior deep brain; CB, cerebellum; FL, frontal lobe; M&P, medulla and pons; PDB, posterior deep brain; PL, parietal lobe; SC, superior colliculi. X, no comparison due to insufficient number of samples (*n* < 6 in at least one of the groups). D, day. CS_mod_, complex shear modulus; L_mod_, loss modulus; S_mod_, storage modulus

	D1			D2			D3			D4		
	S_mod_	L_mod_	CS_mod_	S_mod_	L_mod_	CS_mod_	S_mod_	L_mod_	CS_mod_	S_mod_	L_mod_	CS_mod_
**FL**	0.031			0.007		0.026	0.002	0.038	0.004	x	x	x
**ADB**			0.036	0.029		0.007	< 0.001	< 0.001	< 0.001	0.004	0.004	< 0.001
**PDB**							0.002	0.028	0.002	< 0.001	0.018	< 0.001
**PL**							< 0.001		< 0.001	< 0.001	0.006	< 0.001
**CB**	< 0.001	< 0.001	< 0.001							x	x	x
**M&P**												
**SC**	0.018	0.032	0.018	x	x	x	x	x	x	x	x	x

## Discussion

The potential of measuring brain tissue mechanics for forensic time since death estimations was recently demonstrated [1, 2]. However, the approach showed limited resolution in time since death prediction after 24 h. Screening for notable differences between day 0 (‘fresh samples’) and the subsequent testing days is of paramount importance. Comparisons between different testing days beyond day 0, such as between days 1 and 2, are valuable for detecting points in time with marked biomechanical changes. However, they offer limited practical value for forensic time since death estimations. When storing the brains at 20 °C between death and the mechanical testing, the most prominent differences were observed between fresh samples and samples with a PMI of one day [2]. As a result, day 0 samples could be distinguished from samples with a PMI of at least one day with high sensitivity and specificity [2]. This study used tissue preparation and testing conditions identical to our previous study [2]. The only change was the storage temperature, which was lowered to 4 °C immediately after retrieval of the brains following animal sacrifice.

### Low temperature storage slows down biomechanical changes after death

At a post-mortem storage temperature of 20 °C, the FL, ADB, CB, and SC significantly differed from the day 0 values after one day post-mortem in all investigated biomechanical properties [2]. Three days post-mortem, all brain regions examined in the study significantly differed from the day 0 values at 20 °C in all biomechanical properties [2]. At 4 °C, the biomechanical changes were much slower. Sample storage at 4 °C for one day did not significantly alter the investigated biomechanical properties of brain tissue. This implies that biomechanical analyses for time since death estimations of brain tissue could be delayed for at least 24 h, potentially even 48 h, by maintaining constant cooling at 4 °C. In practice, this could enable forensic investigators to delay biomechanical analyses for at least a couple of hours, for instance, when other tasks must be prioritized. Beyond that, the tissues could be transferred to a specialized laboratory in a moist 4 °C environment if the forensic team on site is unable to perform the biomechanical analyses immediately. However, it remains unclear if the delayed onset of degradation observed only applies when the tissues are transferred to a 4 °C environment in a fresh state or if it also occurs if initiated at a later stage. This question remains to be addressed in future research.

The course of the curves for the investigated biomechanical properties was examined to determine whether general trends apply among the different sampling regions. However, the curves’ progression was region-specific, likely linked to underlying structural differences, including variations in cell types and the white-to-grey matter ratio.

As observed at 20 °C, the CB samples exhibited a distinct drop in the investigated biomechanical properties, followed by a slow decline to reach a plateau [2]. At 4 °C, this significant drop was delayed by one day compared to storage at 20 °C. However, it is important to note that cool storage does not imply a simple one-day time delay for biomechanical changes observed at 20 °C storage. For instance, the FL and ADB samples were among the earliest to differ from day 0 samples after just one day post-mortem at 20 °C [2], but at 4 °C, they were among the more stable sample regions.

### Diagnostic ability of PMIs of at least two days at 4 °C using cerebellar samples

From a practical perspective, forensic investigators require cut-off values to estimate the time since death with high diagnostic ability. Broadly, the diagnostic ability of ROC curves based on the AUC can be categorized as random (AUC: 0.5–0.6), poor (0.6–0.7), fair (0.7–0.8), good (0.8–0.9), and excellent (0.9–1.0) [25]. However, ROC analyses should be interpreted beyond AUC labels [25]. This study revealed that, when using CB samples stored at 4 °C, a PMI of at least two days could be determined with excellent diagnostic ability when reaching CS_mod_ values below 1435 Pa or S_mod_ values below 1313 Pa. A PMI of at least three days could be determined with good diagnostic ability using a L_mod_ cut-off value of 415 Pa for the CB. For a PMI of at least four days, a good diagnostic ability was achieved using a CS_mod_ cut-off value of 1656 Pa for the SC. In conclusion, the hypothesis that cooling at 4 °C extends the value of biomechanical time since death analyses beyond day one after death, compared to storage at 20 °C, can be accepted. However, in contrast to the results at 20 °C, at 4 °C, it was impossible to differentiate samples with a PMI of one day from fresh samples. Therefore, cooling not only extends the value of biomechanical time since death estimations but also lowers the diagnostic value of the method in the very early post-mortem phase, as the biomechanical properties are kept stable. Importantly, the diagnostic abilities stated above require a constant sample temperature for the given intervals. From a practical standpoint, the tissue temperature post-mortem usually varies over time due to different external influences such as exposure to sunlight or the transfer of body parts between places by the perpetrator. Future investigations should explore to what extent the given diagnostic abilities stated here are lowered under varying degrees of temperature variation throughout the given time frames.

Although the diagnostic ability of the CS_mod_ of the SC samples for PMIs of at least four days was good, further analyses are warranted due to the limited number of samples that fell below the cut-off value of 1656 Pa in the given dataset. None of the investigated biomechanical properties of the deep brain samples showed significant differences from day zero within the four testing days post-mortem at a storage temperature of 4 °C. Therefore, future studies should consider extending the analyzed PMI interval of the ADB and PDB at 4 °C to determine their points in time of marked tissue degradation, which are associated with biomechanical changes.

### Temperature as a confounder

Importantly, this study highlighted the temperature sensitivity of the given method. Thus, for biomechanical analyses to be meaningful, knowledge of the temperature curve at the crime scene throughout the past hours to days is crucial. Temperature is a common confounder for forensic time since death estimation and also applies to the established nomogram method of Henssge [26]. Measuring both the ambient temperature and the rectal temperature of the deceased is a standard procedure in every crime scene investigation. Furthermore, local meteorological services can assist in supplying the necessary temperature data to cover the gap between the presumed time of death and the data acquired during the forensic investigation at the scene. When comparing the properties at 4 and 20 °C, the temperature sensitivity was brain region-dependent. It ranged from significant differences throughout all the four testing days in the CS_mod_ values of the ADB samples to no significant differences for any of the tested properties in M&P samples. Especially considering the fair diagnostic ability of M&P samples to determine a PMI of at least three days at 4 °C, its temperature stability between 4 and 20 °C makes it a promising sampling region for biomechanical time since death analyses.

### Outlook

The potential use of biomechanical testing for forensic time since death estimation was demonstrated on ovine tissues. As a next step, the findings should be verified on human tissues. So far, the method has been explored at 4 and 20 °C, representing relevant temperatures from a practical perspective. As it seems to be impractical to test each and every temperature value, interpolation might be an option to fill the gaps after some other relevant temperature values have been investigated, e.g. around the human body temperature. Moreover, different body tissues should be explored applying the here stated protocol as their varying tissue structure and location will cause them to biomechanically respond at different times post-mortem, thereby extending the use of biomechanical analyses for time since death estimations beyond the data stated for brain tissue.

### Limitations

Firstly, this study had limitations in sample population size. However, increasing the population size is unlikely to improve ROC values, which are the major tool for assessing classification potential. Secondly, this study was conducted on ovine tissues. Although the findings provide a thorough starting point for analyses on human samples, the cut-off values should be verified on human tissues to be valid for forensic purposes. Thirdly, differences in brain size and concomitantly in sampling regions may have contributed to the standard deviations of the presented biomechanical properties, as sample harvesting may have slightly varied each time. Fourthly, it was initially intended to use brains only from healthy sheep in this study. However, as the brains were not tested for medical conditions, unknown (veterinary) pathological alterations invisible upon gross inspection might have influenced the investigated biomechanical properties. Fifthly, handling times within the 24-hour intervals might have varied by minutes. Sixthly, the PBS solution used for sample storage between retrieval and testing might have impacted the results, for example, through tissue swelling.

## Conclusions

The storage, loss, and complex shear modulus of eight different ovine brain regions remained stable for at least one day post-mortem at 4 °C. Beyond this point, the biomechanical properties changed in a brain region-specific manner. A post-mortem interval of at least two days could be determined with excellent diagnostic ability using cut-off values of 1435 Pa and 1313 Pa for the complex shear modulus and the storage modulus in cerebellar samples, respectively. However, it is essential to consider the post-mortem temperature as a confounder, recognizing considerable region-specific differences in its impact.

## Data Availability

The data is available on request.
